# Population effectiveness of the pentavalent and monovalent rotavirus vaccines: a systematic review and meta-analysis of observational studies

**DOI:** 10.1186/s12879-017-2613-4

**Published:** 2017-08-15

**Authors:** Daniel Hungerford, Katie Smith, Angela Tucker, Miren Iturriza-Gómara, Roberto Vivancos, Catherine McLeonard, Nigel A Cunliffe, Neil French

**Affiliations:** 1 Institute of Infection and Global Health, University of Liverpool, Liverpool, L69 7BE UK; 2 Field Epidemiology Service, National Infection Service, Public Health England, Liverpool, L1 1JF UK; 3 The Centre for Global Vaccine Research, University of Liverpool, Liverpool, L69 7BE UK; 4 Health Education North West, Liverpool, L3 4BL UK; 5 NIHR Health Protection Research Unit in Gastrointestinal Infections, Liverpool, UK; 6 NIHR Health Protection Research Unit in Emerging and Zoonotic Infections, Liverpool, UK; 7 Department of Medical Microbiology, Alder Hey Children’s NHS Foundation Trust, Liverpool, UK; 8 Royal Liverpool and Broadgreen University Hospitals NHS Trust, Liverpool, UK

**Keywords:** Rotavirus, Vaccine effectiveness, Gastroenteritis, Meta-Analysis, Systematic review

## Abstract

**Background:**

Rotavirus was the leading cause of acute gastroenteritis (AGE) in infants and young children prior to the introduction of routine vaccination. Since 2006 there have been two licensed vaccines available; with successful clinical trials leading the World Health Organization to recommend rotavirus vaccination for all children worldwide. In order to inform immunisation policy we have conducted a systematic review and meta-analysis of observation studies to assess population effectiveness against acute gastroenteritis.

**Methods:**

We systematically searched PubMed, Medline, Web of Science, Cinhal and Academic Search Premier and grey literature sources for studies published between January 2006 and April 2014. Studies were eligible for inclusion if they were observational measuring population effectiveness of rotavirus vaccination against health care attendances for rotavirus gastroenteritis or AGE. To evaluate study quality we use used the Newcastle-Ottawa Scale for non-randomised studies, categorising studies by risk of bias. Publication bias was assessed using funnel plots. If two or more studies reported a measure of vaccine effectiveness (VE), we conducted a random effects meta-analysis. We stratified analyses by World Bank country income level and used study quality in sensitivity analyses.

**Results:**

We identified 30 studies, 19 were from high-income countries and 11 from middle-income countries. Vaccine effectiveness against hospitalization for laboratory confirmed rotavirus gastroenteritis was highest in high-income countries (89% VE; 95% CI 84-92%) compared to middle-income countries (74% VE; 95% CI 67-80%). Vaccine effectiveness was higher for those receiving the complete vaccine schedule (81% VE; 95% CI 75-86%) compared to partial schedule (62% VE; 95% CI 55-69%). Two studies from high-income countries measured VE against community consultations for AGE with a pooled estimate of 40% (95% CI 13-58%; 2 studies).

**Conclusions:**

We found strong evidence to further support the continued use of rotavirus vaccines. Vaccine effectiveness was similar to that reported in clinical trials for both high and middle-income countries. There is limited data from Low income settings at present. There was lower effectiveness against milder disease. Further studies, should continue to report effectiveness against AGE and less-severe rotavirus disease because as evidenced by pre-vaccine introduction studies this is likely to contribute the greatest burden on healthcare resources, particularly in high-income countries.

**Electronic supplementary material:**

The online version of this article (doi:10.1186/s12879-017-2613-4) contains supplementary material, which is available to authorized users.

## Background

Prior to the introduction of rotavirus vaccine into childhood immunisation schedules, rotavirus was the most common cause of severe gastroenteritis in infants and young children. Thus, rotavirus gastroenteritis (RVGE) was estimated to be responsible for 453,000 deaths worldwide in children under 5 years of age in 2008, with over 90% of deaths occurring in low-income countries [1]. The global morbidity from rotavirus infection was also substantial with pre-vaccine introduction studies indicating that approximately 40% of diarrhoeal hospitalisations in children were caused by rotavirus [2]. In middle and high income countries without vaccination the burden of RVGE remains substantial in infants and young children with high rates of disease and RV the major contributor to diarrhoea hospitalisation. In the UK prior to vaccine introduction RVGE was estimated to be responsible for 45% of acute gastroenteritis hospital admissions, 80,000 primary care consultations and 750,000 annual diarrhoeal episodes in children under 5 years of age [3, 4]. In middle income countries such as, Mexico and Peru, prior to vaccine introduction the average incidence of RVGE was 0.3 episodes per child per year in children <2 years, resulting in significant healthcare use and mortality [5]. Although the large majority of severe RVGE occurs among young children, older children and adults can be affected, however rotavirus infection often causes milder symptoms or is asymptomatic in these ages, meaning the true burden and rate of disease incidence is poorly understood.

Since improvements in sanitation and hygiene are not expected to reduce the incidence of rotavirus infection, and treatment of RVGE is limited to rehydration therapy, immunisation of infants is considered the best option for control of the global burden of rotavirus disease. Since 2006 there have been two live-attenuated oral rotavirus vaccines that are licensed for use globally. A two dose monovalent vaccine (Rotarix®, GlaxoSmithKline Biologicals, Belgium), with the first dose typically administered at between 6-8 weeks and a second dose at least 4 weeks later and a three dose pentavalent vaccine (RotaTeq®, Merck), administered at 6-12 weeks of age with subsequent doses at 4-10 week intervals. Randomised controlled trials (RCTs) demonstrated both vaccines to be efficacious against severe RVGE; vaccine efficacy of over 80% has been shown in middle and high-income countries, whilst trials in low-income settings have reported efficacy against severe RVGE of 40-60% [6]. These trials led to a World Health Organisation recommendation for universal vaccination of all children [1, 7]. More than 90 countries have since adopted rotavirus vaccination and the global mortality from RVGE estimated to have fallen to 215 000 in 2013 with almost 50% of deaths occurring in four lower-income countries [8, 9]. Currently within the European Union only nine countries include rotavirus vaccination in their childhood immunisation programme [10, 11].

It is now a decade since the licensing and first introduction of rotavirus vaccination into childhood vaccination schedule. In order to inform immunisation policy, we have conducted a systematic review and meta-analysis of the literature on observational studies in order to assess the population effectiveness of the Rotarix® and RotaTeq® against rotavirus gastroenteritis. Effectiveness was examined by severity and by region.

## Methods

### Inclusion/exclusion criteria

We included prospective or retrospective observational studies (cohort and case-control studies) reporting the population effectiveness of the monovalent Rotarix® (RV1) or pentavalent RotaTeq® (RV5) against healthcare attendance for RVGE or other acute gastroenteritis (AGE), in countries where the vaccines are included in the national immunisation programme or privately offered through medical insurance. Studies published between January 2006 and 28^th^ April 2014 were eligible for inclusion. Review articles, editorials and conference abstracts were included in citation checking but excluded from final analysis. Randomised controlled trials were also excluded.

### Search strategy

We followed the Preferred Reporting Items for Systematic Reviews and Meta-Analyses (PRISMA) guidelines. We systematically searched PubMed, Medline, Web of Science, Cinhal and Academic Search Premier, OpenGrey and the Cochrane Library databases using a well-defined search strategy following a protocol registered on the University of York database for Prospectively Registered Systematic Reviews (PROSPERO: 2014:CRD42014012974). A number of relevant organisations websites were also systematically searched, and included the World Health Organisation, Public Health England, and Centers for Disease Control and Prevention. We systematically searched the literature by, pairing the terms [vacc*] and [rotavirus] with the following key words: [immuni*], [effect*], [ evaluation], and [efficacy].

Authors (DH and CM) replicated the search strategy and independently screened titles and abstracts to identify full studies that were eligible for full publication review. Subsequently these two authors independently assessed the full text publications and their final inclusion was based on a consensus between the reviewers (DH, CM, KS, AT).

### Data extraction

Data extraction was autonomously carried out by three authors (DH, AT, KS) and a collaborator (MSC) using a pre-designed internally piloted extraction tool. For each study the following information was extracted: Author, Year of publication, country and region of study, funding source, study period, country vaccine coverage, study type, sample size, age of subjects, type of vaccine (RV1 and / or RV5) in case and controls groups, case definition, control definition, number of vaccine doses, relative risks / risk ratios (RR) or odds ratio (OR) or vaccine effectiveness (VE) and 95% confidence intervals (95% CI) and, if applicable, a measure of intussusception.

### Grading of selected studies

The Newcastle-Ottawa Scale (NOS) for assessing the quality of non-randomised studies was identified as an appropriate tool to assess study quality [12]. Case-control and cohort-studies were assessed using the tool by the same three researchers that carried out data extraction. To quality assess case-control studies the scale used: 1) adequate definition of a case and the representativeness of cases; 2) controls selection and case definition; 3) matching of controls or adjustment for confounders in analysis; 4) ascertainment of method of cases and controls in terms of exposure (rotavirus vaccination) and non-response rate. To quality assess cohort-studies the factors assessed were: 1) representativeness of the vaccinated cohort and selection of unvaccinated cohort in relation to the vaccinated; 2) ascertainment of vaccination record, confirmation of rotavirus negative at start of study; 3) matching of exposed and non-exposed in design or adjustment for confounders in analysis; 4) ascertainment of outcome (rotavirus infection); 5) follow-up duration in relation to outcome appearance (e.g. 1 year from vaccination date) and was follow-up adequate (we defined adequate as ascertainment of outcomes for >80% of participants). The scale is categorised into three groups, selection, comparability and outcome/exposure; a maximum of nine stars can be awarded to each study. Studies scoring 0 in any of the categories were classified as having a high risk of bias, studies scoring 1 in any categories (moderate risk of bias) and 2 or above in all categories (low risk of bias).

### Statistical analysis

We used Stata, version 14, statistical software (Stata Corp., College Station, TX, USA) to perform all statistical calculations for this meta-analysis. Meta-analyses were conducted separately for cohort and case-control studies. We used the study published RR for cohort studies and OR for case-control studies and calculated standard errors (SE) using study reported confidence intervals in the formula:

$$ SE=\frac{\mathit{\ln}\left( Upper confidence interval\right)-\mathit{\ln}\left( Lower Confidence interval\right)}{3.92} $$

Where studies did not report OR or RR, authors calculated crude OR or RR and SE using reported numbers of cases and controls. When a study reported both unadjusted and adjusted RRs/ORs, adjusted RRs/ORs were included in meta-analysis and unadjusted estimates excluded. Vaccine effectiveness was defined as 100 × (1 − *RR*) or 100 × (1 − *OR*). A random effects model was used to provide pooled estimates of VE. Because of differences in reported vaccine efficacy a decision was taken during data extraction, for analyses to be stratified by country income category, as defined by the World Bank and measured using gross national income per capita [6, 13]. Where a study had reported VE for multiple years the estimate for mid or most recent year (if only two years) were included in the meta-analysis. Heterogeneity was measured using chi-squared (χ2) heterogeneity *p*-values and *I* ^*2*^ statistics. A *p*-value<0.1 was considered to identify statistically significant heterogeneity rather than 0.05 due to the small number of studies included. The percentage of variance across studies due to heterogeneity rather than chance was categorised as low, moderate and high using *I* ^*2*^ values of 25-49%, 50-74% and >=75%, respectively [14].

Sensitivity analysis was conducted based on the NOS score, excluding studies with a high or moderate risk of bias and assessing whether a study was conducted in a country with routine vaccination (part of recommended health policy) or in countries where vaccination provision is private or only available in some states. Subgroup analysis was conducted on: number of doses (1 dose, and full doses), age group, and vaccine type. Both number of doses and vaccine type were identified as important analyses post-hoc. Where studies reported had more than one type of control group the following hierarchy was used to select estimates for use in meta-analyses: 1) community/neighbourhood; 2) hospital non AGE controls; hospital RV negative AGE controls.

Publication bias was checked by funnel plot asymmetry and use of Begg’s test [15].

## Results

### Study characteristics

**Fig. 1:**
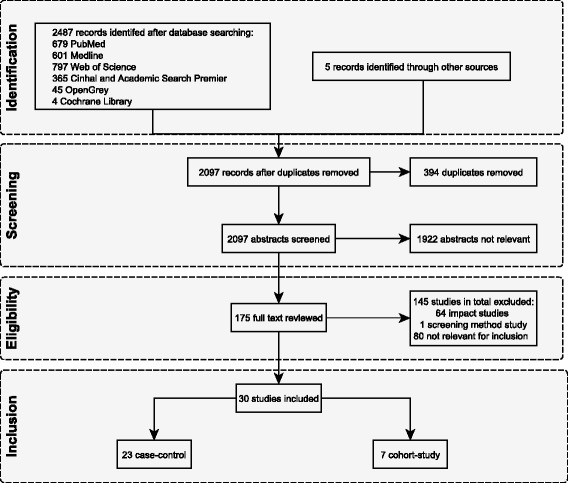
Flow chart of publications included and excluded for this review

The initial search strategy identified 2,097 studies as potentially relevant; of these, 30 were eligible for inclusion in the review (Fig. 1) [16–45]. Two summary tables of study characteristics are available in Additional file 1. Seven studies were cohort studies and 23 were case-control studies (Tables 1 and 2.) Nineteen were from high-income countries and eleven were from middle income countries. Seven studies declared some funding from industry related to the rotavirus vaccines under study. Over a third of studies were conducted in the USA (n=12). The majority of studies (27/30) reported on RVGE hospitalisations and/or emergency department (ED) attendances for AGE with a positive laboratory test for rotavirus. A study by Mast et al., [16] measuring vaccine effectiveness against RVGE hospitalisation and ED attendances included only cases with severe disease defined by a Vesikari score of greater than 11 in their VE estimate [16, 46, 47]. Two studies reported RVGE ED attendances and hospitalisations combined. Five studies included community consultations for either RVGE or AGE [17–21], which included a range of definitions including outpatient attendance, physician consultation and telephone consultation. The study by Fontes Vieira et al. 2011 conducted in Brazil on a community cohort examined effectiveness of vaccination against laboratory confirmed RVGE but did not report an estimate of VE; a crude estimate was therefore calculated by the authors [17]. In the majority of studies, laboratory confirmation of RVGE followed hospitalisation, an ED attendance or GP consultation for gastroenteritis symptoms such as diarrhoea. Study selection identified five studies from countries (Spain and Israel) where routine childhood vaccination is not available but either the monovalent or pentavalent vaccine is available privately and / or only in some states [18, 22, 24, 31, 42].

**Table 1:** Cohort study vaccine effectiveness against hospitalisations, emergency department attendances and community consultations for RVGE or AGE

Study	Country	Vaccine	Age-group (months)	Cohort year	Vaccinated (N)		Incidence among unvaccinated (N)	Vaccine effectiveness (95% CI)	
					Dose	Incidence		Unadjusted	Adjusted
Hospitalisation for RVGE									
Eberly et al. 2011 [23]	USA	RV1 and RV5	<60	All	1	NR	581/237660	86 (78-91)	NR
					1 or more	42/140213		88 (83-91)	NR
					2 RV1 / 3 RV5	11/NR		90 (82-95)	NR
Panozzo et al. 2014* [30]	USA	RV1 and RV5	8-20	1	1 or more	3/68380	60/64929	NR	87 (58-96)
				2		23/175890	91/91051	NR	87 (80-92)
				3		22/250035	74/61218	NR	92 (87-95)
				4		8/254377	13/41946	NR	90 (75-96)
Hospitalisation and ED attendance for RVGE									
Wang et al. 2010 [20]	USA	RV5	<36	All	3	0/7700	23/5831	100 (87-100)	NR
Wang, 2013 [21]	USA	RV5	<36	All	1	2/5019	11/3343	88 (45-99)	NR
				All	2	1/5886	13/4432	94 (61-100)	NR
Hospitalisation and ED attendance for AGE									
Wang et al. 2010 [20]	USA	RV5	<36	All	3	87/7700	160/5831	59 (46-69)	59 (47-68)
Wang, 2013 [21]	USA	RV5	<36	All	1	53/5019-	63/3343	44 (18-62)	46 (22-63)
				All	2	78/5886	98/4432	40 (18-56)	39 (16-55)
Hospitalisation for AGE									
Panozzo et al. 2014* [30]	USA	RV1 and RV5	8-20	1	1 or more	142/68378	271/64928	NR	22 (3-37)
				2		413/175765	317/90882	NR	40 (30-48)
				3		512/249838	300/61136	NR	56 (49-62)
				4		398/254232	109/41888	NR	41 (27-53)
Community consultations for RVGE									
Wang et al. 2010 [20]	USA	RV5	<36	All	3	1/7700	20/5831	96 (76-100)	NR
Wang et al. 2013 [21]	USA	RV5	<36	All	1	0/5019 -	7/3343	100 (54-100)	NR
				All	2	4/5886	5/4432	40 (<0-88)	NR
Community consultations for AGE									
Fontes-Vieira et al. 2011 [17]**	Brazil	RV1	<12	1	2	87/100	84/100	-4 (-16 to 8)	NR
				2		52/100	42/100	-24 (-67 to 8)	NR
Muhsen et al. 2011 [18]	Israel	RV1	<12	All	1	153/716	8801/18591	54 (47-60)	NR
				All	2	1605/6870		50 (47-52)	NR
Nolan et al. 2012 [19]†	USA	RV5	<24	1	1 or more	NR	NR	NR	28 (-21 to58)
				1		NR	NR	NR	22 (-13 to 46)
				2		NR	NR	NR	37 (-37 to 71)
Wang et al. 2010 [20]	USA	RV5	<36	All	3	1321/7700	1377/5831	27 (22-33)	28 (22-33)
Wang et al. 2013 [21]	USA	RV5	<36	All	1	651/5019	521/3343	17 (6-26)	17 (7-26)
				All	2	774/5886	847/4432	31 (24-38)	28 (21-35)

* Direct effect estimates, ** Crude VE calculated by authors, *NR* not reported, *ED* emergency department, *d* days, *AGE* acute gastroenteritis, *RVGE* rotavirus gastroenteritis, †GP consultations reported, paper also reported telephone triage and episodes (calls and visits within ten days), two cohorts were followed, the 1^st^ for two seasons and 2^nd^ for one season

**Table 2:** Case-control study vaccine effectiveness against hospitalisations, emergency department attendances for RVGE or AGE (partial is for 1 dose of RV1 or RV5 if reported)

Study	Country	Vaccine	Age in months (m) and / or days (d)	Control group	Cases vaccinated (N/Total)		Controls vaccinated (N/Total)		Vaccine effectiveness (95% CI)	
					Partial	Full	Partial	Full	Partial	Full
Hospitalisations for RVGE										
Braeckman et al. 2012 [32]	Belgium	RV1	<36	Hospital - RV (-) AGE	81/179*	70/160	208/228	179/198	91 (82-95)	90 (81-95)
			3-11		36/77*	30/66	99/104	79/84	93 (80-97)	91 (75-97)
			12-31		45/102*	40/94	109/124	100/114	89 (75-95)	90 (76-96)
Carvalho-Costa et al. 2009 [25]	Brazil	RV1	<60	Hospital - RV (-) AGE	NR	4/14	NR	35/60	NR	71 (-15-93)
Castilla et al. 2012 [31]	Spain	RV1 & RV5	3-59	Hospital - RV (-) AGE	NR	9/262	NR	80/518	NR	83 (65-93)
			<24		NR	8/215	NR	69/371	NR	82 (60-93)
			24-59		NR	1/43	NR	11/99	NR	89 (17-99.8)
Correia et al. 2010 [35]	Brazil	RV1	6-11	Hospital - RV (-) AGE	NR	NR	NR	NR	NR	77 (42-91)
Cortese et al. 2011 [36]	USA	RV5	>7	Hospital - RV (-) AGE	NR	20/140	NR	163/280	NR	92 (86-96)
			>7	Community_a_	NR	17/221	NR	672/1953	NR	90 (84-94)
Cortese et al. 2013 [37]	USA	RV1	>7	Hospital - RV (-) AGE	NR	2/30	NR	101/140	NR	98 (90-100)
			>7	Community_a_	NR	2/28	NR	206/440	NR	94 (71-99)
Cotes-Cantillo et al. 2014 [29]	Colombia	RV1	<60	Hospital - RV (-) AGE	NR	6/84	NR	670/711	NR	-2 (-182 to 63)
			6-11		NR	12/15	NR	628/655	NR	84 (23-97)
			≥12		NR	64/67	NR	628/655R	NR	-80 (-559 to 51)
de Palma et al. 2010 [38]	El Salvador	RV1	<25	Community	72/171	152/251	199/352	617/770	51 (26-67)	76 (64-84)
			6-11		NR	49/63	NR	205/222	NR	83 (68-91)
			12-24		NR	79/108	NR	284/335	NR	59 (27-77)
Desai et al. 2010 [39]	USA	RV1 & RV5	2-35	Hospital - RV (-) AGE	5/42*	NR	24/80	NR	94 (55-99)	NR
				Community	5/42*	NR	21/73	NR	96 (59-99.8)	NR
Guh et al. 2011 [40]	USA	RV5	<36	Community	2/54	0/54	34/270	25/270	84 (25-96)	92 (48-100)
Ichihara et al. 2014 [41]	Brazil	RV1	4-24	Hospital – RV (-) and non-vaccine preventable	33/215	115/215	279/1961	1481/1961	60(37-75)	72(44-85)
Justino et al. 2011 [33]*	Brazil	RV1	3-11	Hospital - non AGE or vaccine preventable	NR/120	NR/77	NR/120	NR/77	61 (28-78)	56 (12-78)
			12-35		NR/324	NR/235	NR/324	NR/235	35 (4-56)	32 (-4 to 46)
			3-35		NR/444	NR/312	NR/444	NR/312	44 (23-60)	40 (14-58)
			3-11	Community no AGE symptoms	NR/91	NR/64	NR/91	NR/64	89 (63-97)	96 (68-99)
			12-35		NR/240	NR/185	NR/240	NR/185	48 (17-68)	65 (37-81)
			3-35		NR/331	NR/249	NR/331	NR/249	62 (42-75)	76 (58-86)
Muhsen et al. 2010 [42]*	Israel	RV1 & RV5	<28 (approx.)	Hospital - RV (-) AGE	2/111	NR	36/216	NR	89 (52-98)	NR
Martinon-Torres et al. 2011 [24]	Spain	RV1 & RV5	<24	Hospital - RV (-) AGE	2/75	1/74	22/186	130/294	80 (11-95)	98 (87-99.8)
Patel et al. 2009 [26]	Nicaragua	RV5	<24	Community	31/80	143/192	116/213	442/539	52 (14-73)	43 (9-64)
				Hospital - RV (-) AGE	31/80	143/192	106/181	350/425	60 (24-78)	49 (17-68)
				Community and Hospital - RV (-) AGE	31/80	143/192	222/394	792/964	55 (22-74)	44 (15-63)
Patel et al. 2013 [43])	Bolivia	RV1	6-11	Hospital - RV (-) non AGE	NR	NR	NR	NR	NR	77 (51-89)
			12-35		NR	NR	NR	NR	NR	76 (59-86)
			<36		100/192	208/300	226/343	857/974	56 (32-72)	77 (65-84)
			6-11	Hospital - RV (-) AGE	NR	NR	NR	NR	NR	64 (34-80)
			12-35		NR	NR	NR	NR	NR	72 (52-86)
			<36		100/192	208/300	131/208	510/587	36 (0-59)	69 (54-79)
Patel et al. 2012 [27]	Nicaragua	RV5	6-44	Hospital - RV (-) Non AGE or vaccine preventable and Community	NR	773/849	NR	3914/4062	NR	70 (59-78)
			6-11		NR	NR	NR	NR	NR	73 (54-84)
			12-44		NR	NR	NR	NR	NR	68 (51-79)
			6-44	Hospital - RV (-) AGE	NR	773/849	NR	3097/3247	NR	45 (25-59)
			6-11		NR	NR	NR	NR	NR	64 (43-78)
			12-44		NR	NR	NR	NR	NR	30 (-5 – 53)
Payne et al. 2013 [28]	USA	RV1	<60	Hospital - RV (-) AGE	NR/22	NR	NR/34	NR	32 (-156-82)	NR
		RV5			NR/130	NR	NR/372	NR	86 (74-91)	NR
Snelling et al. 2009 [44]	Australia	RV1	<60	Community	10/21	3/21	58/83	32/83	57 (<0-83)	85 (23-97)
Staat et al. 2011 [34]	USA	RV5	1-37	Hospital - RV (-) AGE	1/38	3/40	12/44	17/49	89 (16-99)	95 (48-99)
			1-37	Hospital - RV (-) ARI	1/60	29/102	5/64	40/113	94 (55-99)	82 (50-93)
Hospital admissions for AGE										
Snelling et al. 2009 [44]	Australia	RV1	<60	Community	21/42	11/42	120/166	72/166	67 (29-84)	78 (40-92)
ED attendances for RVGE										
Cotes-Cantillo et al. 2014 [29]	Colombia	RV1	<60	ED - RV (-) AGE	NR	143/156	NR	670/711	NR	16 (79-61)
			6-11		NR	27/31	NR	27/655	NR	79 (24-94)
			≥12		NR	112/119	NR	628/655	NR	-40 (-271 to 47)
Cortese et al. 2011 [36]	USA	RV5	>7	Hospital- RV (-) AGE	NR	8/41	NR	163/280	NR	81 (53-92)
			>7	Community	NR	6/62	NR	138/567	NR	84 (58-94)
Cortese et al. 2013 [37]	USA	RV1	>7	Hospital- RV (-) AGE	NR	20/65	NR	101/140	NR	86 (67-94)
			>7	Community	NR	17/61	NR	438/862	NR	65 (35-81)
Payne et al. 2013 [28]	USA	RV1	<60	ED- RV (-) AGE	38	NR	121	NR	78 (46-91)	NR
		RV5	<60	ED- RV (-) AGE	229	NR	1439	NR	81 (70-84)	NR
Staat et al. 2011 [34]	USA	RV5	15 d-47m	ED- RV (-) AGE	5/56	8/59	12/66	22/76	75 (-64 -96)	74 (16-92)
			15 d-47m	ED - ARI	7/76	8/77	16/101	54/139	45 (-80 -83)	88 (64-96)
Hospitalisations and ED attendances for RVGE										
Cortese et al. 2011 [36]	USA	RV5	>7	Hospital RV (-) AGE	10/163	23/283	20/137	141/341	69 (27-87)	89 (81-94)
			8-11		NR	4/24	NR	63/92	NR	93 (75-99)
			12-23		NR	21/102	NR	86/147	NR	89 (77-94)
			>23		NR	3/55	NR	14/41	NR	91 (62-99)
			56d-5m		11/69	NA	97/248	NA	71 (40-87)	NA
			56d-5m	Community_a_	11/69	NA	184/633	NA	62 (20-82)	NA
			>7		NR	23/283	NR	850/2520	NR	89 (83-93)
			8-11		NR	3/109	NR	194/1009	NR	94 (78-99)
			12-23		NR	17/116	NR	517/988	NR	88 (80-93)
			>23		NR	3/58	NR	139/523	NR	87 (56-96)
Cortese et al. 2013 [37]	USA	RV1	>7	Hospital RV (-) AGE	NR	22/95	NR	101/140	NR	91 (80-95)
			8-11		NR	5/14	NR	45/61	NR	85 (35-97)
			12-23		NR	14/66	NR	46/68	NR	91 (75-96)
			>7	Community_a_	NR	19/89	NR	644/1302	NR	76 (58-86)
			8-11		NR	4/14	NR	114/196	NR	70 (24-91)
			12-23		NR	13/65	NR	462/967	NR	76 (53-87)
Donauer et al. 2013 [45]	USA	RV5	<36	Community	8/76	2/76	165/743	329/743	77 (14-94)	92(60-99)
				Hospital - RV (-) AGE	8/76	2/76	47/179	15/179	68 (-18 to 91)	92(21-99)
				Hospital - RV (-) ARI	8/76	2/76	71/288	27/288	58 (-38 to 87)	92(33-99)
Mast et al. 2011 [16]	Nicaragua	RV5	Overall	Community	NR	241/300	NR	812/851	NR	87(74-93)
			<12		NR	62/84	NR	219/225	NR	93(62-99)
			≥ 12		NR	179/216	NR	593/626	NR	85(69-93)
			Overall	Hospital – RV(-) AGE	NR	241/300	NR	711/792	NR	64(44-78)
			<12		NR	62/84	NR	215/233	NR	78(49-91)
			≥ 12		NR	179/216	NR	496/559	NR	55(22-74)
			Overall	Combined	NR	241/300	NR	1523/1643	NR	76(63-84)
			<12		NR	62/84	NR	434/458	NR	85(66-93)
			≥ 12		NR	179/216	NR	1089/1185	NR	71(51-82)
Payne et al. 2013 [28]	USA	RV1	<60	Hospital - RV (-) AGE	46	56	83	140	57 (-45-87)	70 (39-86)
			<60 (2011 season only)		53	NR	96	-	64 (23-83)	NR
			12-23		7	NR	54	-	56 (-59-100)	NR
			24-35		46	NR	79	NR	86 (60-95)	NR
		RV5	<60	Hospital - RV (-) AGE	233	537	307	1445	70 (50-82)	84 (78-88)
			<60 (2010 season only)		111	NR	924	NR	82 (69-89)	NR
			<60 (2011 season only)		248	NR	887	NR	84 (77-89)	NR
			12-23		34	NR	402	NR	85 (63-94)	NR
			24-35		121	NR	681	NR	89 (82-93)	NR
			36-47		91	NR	414	NR	83 (69-90)	NR
Staat et al. 2011 [34]	USA	RV5	15d -37m	Hospital - RV (-) AGE	9/136	16/143	40/191	87/238	74 (37-90)	87 (71-94)
			1-11		6/65	3/62	30/109	19/98	63 (7-87)	86 (31-97)
			12-23		3/38	8/43	6/45	48/87	81 (22-97)	90 (65-97)
			15d -37m	Hospital - RV (-) ARI	10/159	17/166	79/391	195/507	73% (43-88)	85 (72-91)
			1-11		6/76	3/73	60/230	43/213	74 (31-90)	84 (41-96)
			12-23		4/41	9/46	15/87	104/176	70 (16-92)	87 (68-95)
Any episode of RVGE										
Bellido-Blasco et al. 2012 [22]	Spain	RV1 & RV5	<36	RV (-) AGE	2/71	NR	57/261	NR	88 (46–99.7)	NR
Castilla et al. 2012 [31]	Spain	RV1 & RV5	3-59	RV (-) AGE	45/756*	34/756	1094/6036*	849/6036	78 (70-84)	78 (68-85)
			3-11		NR	12/309	NR	248/1365	NR	78 (58-88)
			12-23		NR	16/318	NR	481/2678	NR	82 (69-89)
			24-59		NR	6/118	NR	120/1748	NR	61 (0-84)
Martinon-Torres et al. 2011 [24]	Spain	RV1 & RV5	<24	RV (-) AGE	3/143	8/148	22/186	130/294	84(46-95)	93 (85-97)

AGE, all cause gastroenteritis symptoms; ARI, acute respiratory infection; RV (-), rotavirus test negative; ED, emergency department; d, days

*One or more doses reported for partial

Controls for case-control studies were primarily hospital controls that were admitted for AGE symptoms but with a rotavirus negative laboratory test result. A few studies also used community asymptomatic controls or non-AGE hospital controls, such as children admitted with acute respiratory infection (Additional file 1: Table S2). VE was measured for a range of age groups across studies.

### Quality of included observational studies

The quality of studies varied considerably (Table 3). It was difficult to ascertain for most studies whether history of disease in control subjects was considered. The majority of studies used a combination of vaccination cards and medical records to ascertain vaccination status. The majority of studies either matched controls or adjusted for age in the analysis as a minimum and those with community controls often used an indicator of residence such as GP location as a covariate. A high risk of bias was identified in two out of seven cohort studies [17, 23] and two out of 23 case-control studies had a high risk of bias [24, 25].

**Table 3:** Quality of observational studies included in the review of rotavirus vaccine effectiveness. Using the Newcastle-Ottawa Scale

Study No.	Study	Country	Study design	Selection	Comparability	Outcome / exposure	Overall	Bias
1	Bellido-Blasco et al. 2012 [22]	Spain	Case-control	3	2	3	8	Low
2	Braeckman et al. 2012 [32]	Belgium	Case-control	3	2	3	8	Low
3	Carvalho-Costa et al. 2009 [25]	Brazil	Case-control	2	0	1	3	High
4	Castilla et al. 2012 [31]	Spain	Case-control	3	2	3	8	Low
5	Correia et al. 2010 [35]	Brazil	Case-control	3	1	3	8	Moderate
6	Cortese et al., 2011 [36]	USA	Case-control	3	2	3	8	Low
7	Cortese et al. 2013 [37]	USA	Case-control	3	2	3	8	Low
8	Cotes-Cantillo et al. 2014 [29]	Colombia	Case-control	3	2	3	8	Low
9	de Palma et al. 2010 [38]	El Salvador	Case-control	3	2	3	8	Low
10	Desai et al. 2010 [39]	USA	Case-control	3	2	2	7	Low
11	Donauer et al. 2013 [45]	USA	Case-control	2	2	2	6	Low
12	Guh et al. 2011 [40]	USA	Case-control	4	1	3	9	Moderate
13	Ichihara et al. 2014 [41]	Brazil	Case-control	3	2	2	7	Moderate
14	Justino et al. 2011 [33]	Brazil	Case-control	4	2	3	9	Low
15	Martinon-Torres et al. 2011 [24]	Spain	Case-control	4	0	2	6	High
16	Mast et al. 2011 [16]	Nicaragua	Case-control	3	2	1	7	Moderate
17	Muhsen et al. 2010 [42]	Israel	Case-control	3	2	1	6	Moderate
18	Patel et al. 2009 [26]	Nicaragua	Case-control	4	2	3	9	Low
19	Patel et al. 2012 [27]	Nicaragua	Case-control	4	2	2	8	Low
20	Patel et al. 2013 [43]	Bolivia	Case-control	3	2	3	8	Low
21	Payne et al. 2013 [28]	USA	Case-control	3	2	2	7	Low
22	Snelling et al. 2009 [44]	Australia	Case-control	3	2	3	8	Low
23	Staat et al. 2011 [34]	USA	Case-control	3	2	2	7	Low
24	Eberly et al. 2011 [23]	USA	Cohort	3	0	3	6	High
25	Fontes-Vieira et al. 2011 [17]	Brazil	Cohort	3	0	2	5	High
26	Muhsen et al. 2011 [18]	Israel	Cohort	3	1	3	7	Moderate
27	Nolan et al. 2012 [19]	USA	Cohort	4	2	3	9	Low
28	Panozzo et al. 2014 [30]	USA	Cohort	4	2	3	9	Low
29	Wang et al. 2010 [20]	USA	Cohort	4	1	3	8	Moderate
30	Wang et al. 2013 [21]	USA	Cohort	4	1	3	8	Moderate

### Meta-analysis of vaccine effectiveness against hospitalisation or combined hospitalisation and emergency department attendance for laboratory confirmed RVGE

**Fig. 2:**
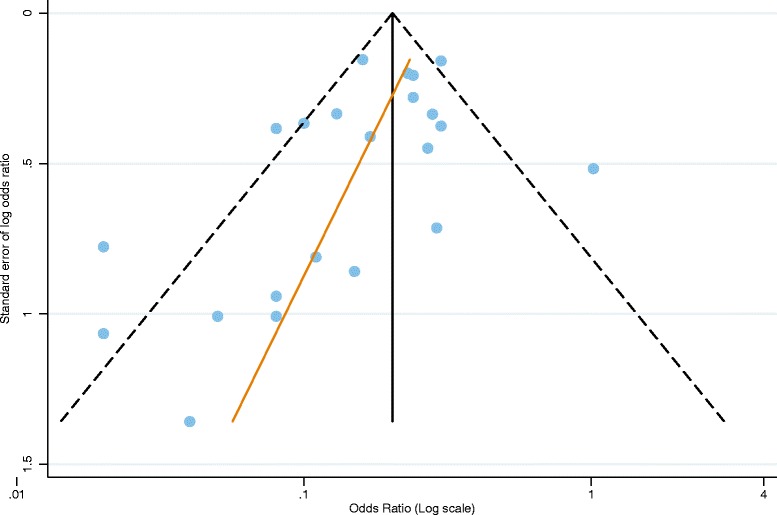
Funnel plot of vaccine effectiveness against hospitalisation or hospitalisation and emergency department attendance for laboratory confirmed RVGE (only adjusted effect estimates included)

For this outcome measure cohort studies were too few to conduct a meta-analysis (Table 1). We therefore, included 21 out of the 22 case control studies that measured vaccine effectiveness against hospitalisation or hospitalisation and ED attendance for laboratory confirmed RVGE in the meta-analysis. Patel et al. 2009 was excluded because a more recent publication Patel et al., 2012 provided more recent estimates of effectiveness for the same cohort [26, 27]. If studies reported more than one age group the overall estimate for the broadest age group was included in the meta-analysis. Studies which reported on 0 doses vs full dose or 0 dose vs 1+ dose were included. Some 22 estimates from 21 studies were included, as Payne et al, 2013 had separate results for RV1 and RV5 [28]. The funnel plot shows some asymmetry, however this is not significant for Begg’s (*p*=0.06) tests (Fig. 2). Therefore, we included all 21 studies in the meta-analysis.

**Fig. 3:**
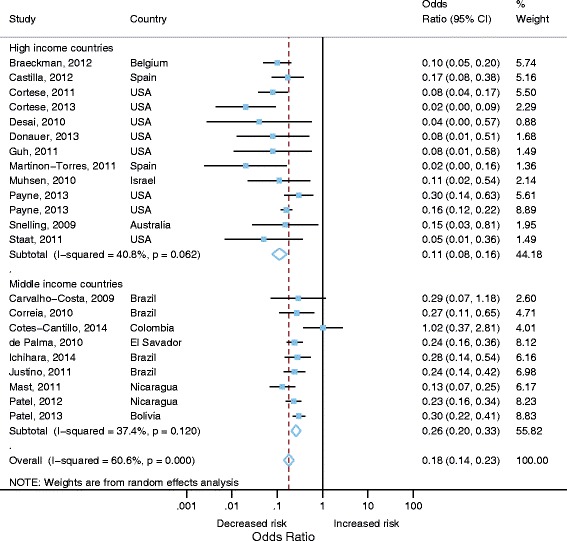
Vaccine effectiveness against hospitalisation or hospitalisation and emergency department attendance for laboratory confirmed RVGE (only adjusted effect estimates included)

There was statistically significant heterogeneity (*I* ^2^= 60.6, *p* < 0.001) across all studies (Fig. 3). Therefore pooled estimates of OR (0.18, 95% CI 0.14–0.23, 21 studies [22 estimates], *p*<0.001) were calculated using a random effects model. The pooled VE was therefore 82% (95% CI 80–88%; 21 studies [22 estimates], *p*<0.001). A stratified analysis by World Bank Country Classifications calculated pooled estimated ORs. Pooled VE was lower in middle-income countries (74% VE; 95% CI 67-80%; 9 studies, *p*<0.001) compared with high income countries (89% VE; 95% CI 84-92%; 12 studies [13 estimates], *p*<0.001). There was low to moderate heterogeneity for middle (*I* ^2^= 37.4%, *p*=0.120) and high income countries (*I* ^*2*^ = 40.8%, *p*=0.06). The study by Cotes-Cantillo et al., 2014 was the only study to report a negative vaccine effectiveness (-2 VE; 95% CI -182 to 63%) [29].

Pooled estimates for case-control studies in high income settings (89% VE; 95% CI 84-92%; 12 studies [13 estimates]) were comparable to the three unpooled cohort study estimates. All cohort studies were conducted in high income settings. One study reported an adjusted VE estimate of 87% (95% CI 80-92%) and other two studies stated unadjusted estimates of 100% (95% CI 87-100%), and 90% (95% CI 82-95%) [20, 23, 30].

#### Sensitivity analysis

We identified the possibility of study bias using the Newcastle-Ottawa Scale. The pooled OR for studies with a low risk of bias was 0.18 (95% CI 0.13-0.25; 13 studies [14 estimates], *p*<0.001), suggesting that any bias in these studies may have been minimal. The corresponding pooled estimates by World Bank classification did not change substantially. When studies from countries with state-based or private rotavirus vaccine provision (Castilla et al 2012, Martinon-Torres, 2011 and Muhsen et al., 2010) were dropped from the meta-analysis the pooled OR remained similar (0.19 OR; 95% CI 0.14-0.25; 18 studies [19 estimates], *p*<0.001) [24, 31, 42].

To further investigate the potential for publication bias, specifically that the effect of industry funding, we excluded 4 studies which had measured RVGE hospitalisations but were funded by industry [16, 32–34]. Overall pooled ORs remained comparable (0.19 OR; 95% CI 0.14-0.26 ; 17 studies [18 estimates], *p*<0.001). We also established that including an estimate from a study which measured vaccine effectiveness against severe disease (Vesikari score ≥11), did not cause an overestimation of the pooled estimate [16].

#### Subgroup analyses

##### Age

**Fig. 4:**
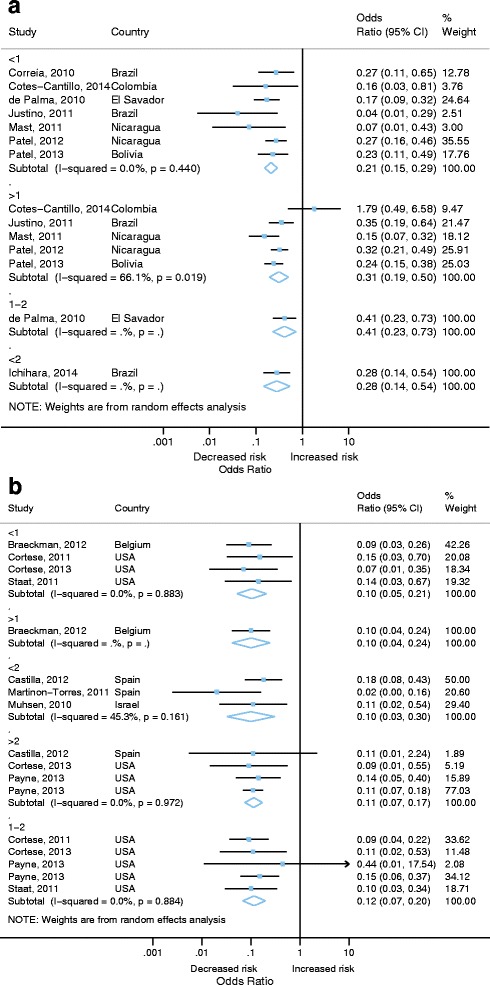
Vaccine effectiveness against hospitalisation or hospitalisation and emergency department attendance for laboratory confirmed RVGE comparing partial age groups **a** middle income countries, **b** high income countries. (only adjusted effect estimates included)

Because the age groups included in studies were varied we grouped ages in to the following groups to assess VE by crude age groups: <1 year, <2 years, >1 year and 1-2 years of age. In middle-income countries there was some variation in pooled estimates by age group but confidence intervals overlapped between estimates (Fig. 4a). Here estimates should be interpreted with caution as there was only 1 study in each of the 1-2 year and <2 year groups. Additionally, the VE estimate for children aged >12 months from the 2014 study by Cotes-Cantillo stood out as being heterogeneous. As the authors note this is likely to be due to the low sample size in this group and potentially due to a variation in strain dominance by age [29]. Estimates were very similar across age ranges in high-income countries (Fig. 4b).

##### Full v partial vaccine dose

**Fig. 5:**
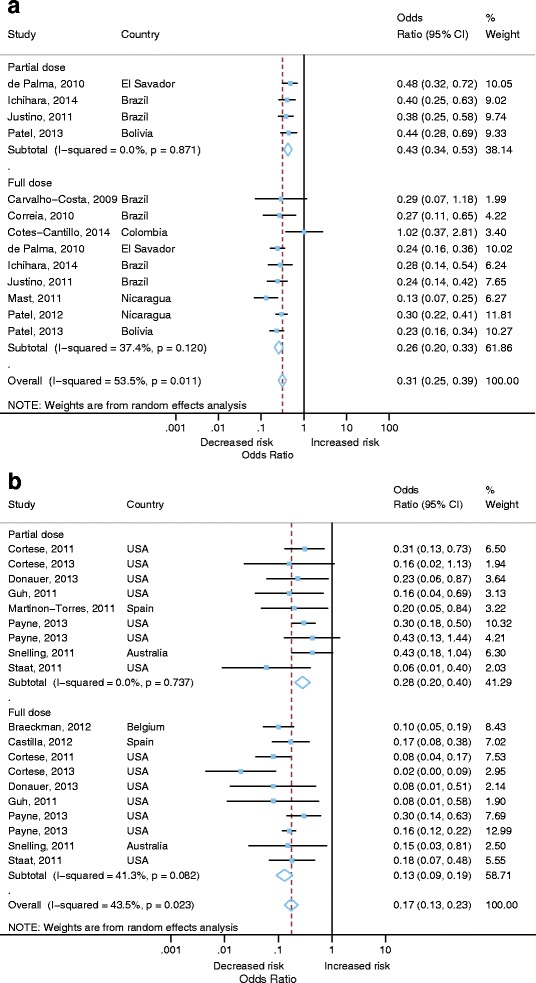
Vaccine effectiveness against hospitalisation or hospitalisation and emergency department attendance for laboratory confirmed RVGE comparing partial dose to full dose **a** middle income countries, **b** high income countries (only adjusted effect estimates included)

To determine the impact of the number of vaccine doses on vaccine effectiveness we compared studies which reported full dose vaccination with first dose vaccination for RV1 and RV5 vaccine effectiveness estimates. There were 21 studies which either reported full or partial vaccination giving 32 estimates (13 partial schedule; 19 full vaccine schedule). Pooled vaccine effectiveness for full dose (81% VE; 95% CI 75-86%, *p*<0.001) was higher than for partial dose (62% VE; 95% CI 55-69%, *p*<0.001). However, there was moderate to high heterogeneity for studies reporting estimates for full dose (I^2^= 60.2%, *p*<0.001). When stratified by World Bank classification, this difference was most pronounced in middle income countries, where pooled vaccine effectiveness for full dose was 74% (95% CI 67-80%, *p*<0.001) and 57% (95% CI 47-66%, *p*<0.001) for partial dose (Fig. 5a). Wider confidence intervals were reported in studies reporting full vaccination, likely due to smaller available sample sizes. In high income countries VE for partial vaccine dose was 72% (95% CI 60-80%, *p*<0.001) compared to 87% (95% CI 81-91%, *p*<0.001) for full dose (Fig. 5b).

##### RV1 and RV5 vaccine effectiveness

Pooled VE for RV1 and RV5 stratified by World Bank country classification showed that RV5 has slightly higher VE point estimates in both high and middle income countries but this difference was not significant. RV1 is the predominant vaccine used in studies from middle income countries included in the meta-analysis (7/10). In high income countries three studies reported VE for RV1, six for RV5 and four RV1 and RV5 combined.

### Meta-analysis of vaccine effectiveness against emergency department attendances for RVGE

**Fig. 6:**
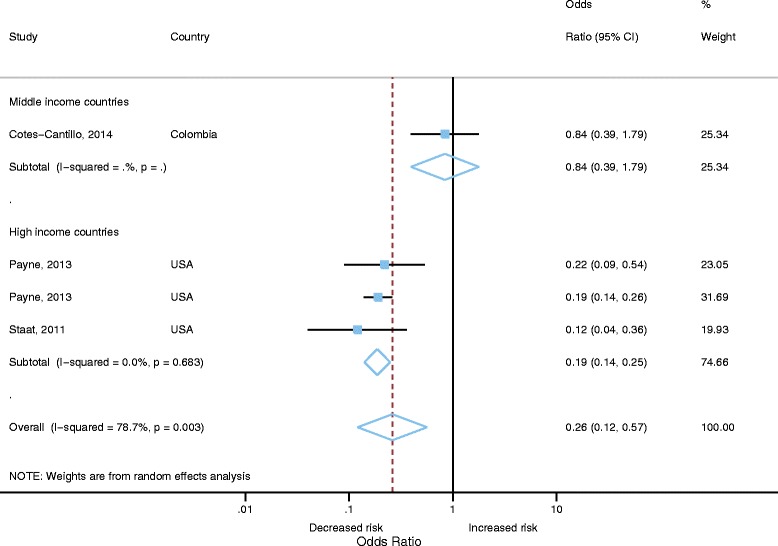
Vaccine effectiveness against emergency department attendances for RVGE (only adjusted effect estimates included)

There were 4 estimates from three studies that included measure of VE for ED attendances for RVGE [28, 29, 34]. Three estimates were from high income countries and one from middle-income. Publication bias was not assessed as there were inadequate numbers of studies to properly assess via a Begg’s. Heterogeneity was high for these studies (I2=78.7%, *p*<0.001). Random effects meta-analysis gave a pooled OR of 0.26 (95% CI 0.12-0.57, *p*=0.001), indicating a significant effect of vaccination against ED attendances for RVGE (Fig. 6). Analysis stratified by World Bank country classifications showed significant VE of 81% (95% CI 75-86%, *p*<0.001) for studies from high income countries. There was only one study from middle income countries with a VE of 16% (95% CI -79% to 61%, *p*=0.651).

### Meta-analysis of vaccine effectiveness against community consultations for AGE

**Fig. 7:**
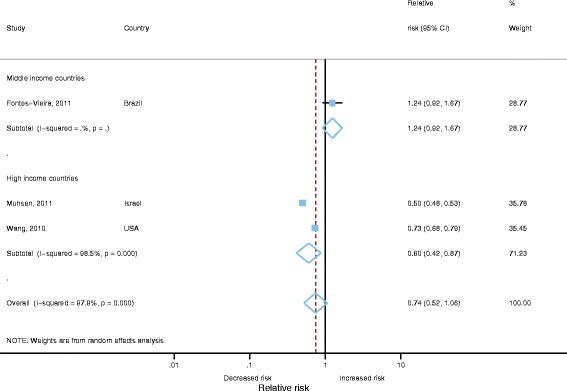
Vaccine effectiveness against community consultations for AGE (only unadjusted effect estimates included)

A single study reported full dose vaccine effectiveness against community consultations for laboratory confirmed RVGE, reporting a high unadjusted VE estimate of 96% (95% CI 76-100%) [20, 21]. However, four cohort studies included estimates of vaccine effectiveness for community consultations for AGE [17–21]. Three studies were from high income countries and one from middle-income. Only two out of the four studies reported adjusted estimates. The study by Nolan et al., 2012 only reported an adjusted VE for 1 or more doses [19]. We therefore could only include three cohort studies which report unadjusted VE in the meta-analysis. Publication bias was not assessed as there were inadequate numbers of studies to properly assess via a Begg’s test. Heterogeneity was very high for these studies (I^2^=97.9%, *p*<0.001). Random effects meta-analysis gave a pooled RR of 0.74 (95% CI 0.52-1.06, *p*=0.10), indicating a non-significant effect of vaccination against community consultations for AGE (Fig. 7). However, analysis stratified by World Bank country classifications showed significant VE of 40% (95% CI 13-58%, *p*=0.008) for studies from high income countries. There was only one study from middle income countries with a VE of -24% (95% -67% to 8%, *p*=0.157), this study was assessed as having a high risk of bias as crude VE was calculated by the authors and children in the vaccinated cohort were significantly younger than the unvaccinated cohort.

## Discussion

The pooled data from case-control studies indicates that RV vaccination is highly effective for preventing hospitalisations and / or ED attendances for laboratory confirmed rotavirus, as VE was 89% (95% CI 84-92) for high income countries and 74% (95% CI 67-80) for middle income countries. This finding is further supported by comparable estimates from the unpooled cohort studies. Most studies reporting VE for community consultations could not identify the causative organism and therefore reported a much lower effectiveness against community consultations for AGE (26% VE; 95% CI -6 to 48%). However, like VE against RVGE hospitalisations and ED attendances, the pooled VE for community consultations for AGE was significant in high income countries (40% VE; 95% CI 13-58%). This study was also able to show that studies from countries with similar economic classification demonstrate similar effectiveness and that effectiveness was lower in middle income countries compared with high income countries. Vaccine effectiveness against RVGE hospitalisations in high income countries (89% VE; 95% CI 84-92%) was consistent with but at the upper limit of that reported in reported in a Cochrane Review of RCTs; 85% (95% CI 80-88%) for RV1 and 82% (95% CI 50-93%) for RV5 for preventing severe rotavirus diarrhoea in children up to two years of age [6]. Differing study definitions of severity and age maybe responsible for the slight difference between pooled estimates. Additionally, pooled VE estimates from meta-analysis for RVGE hospitalisations in middle income countries (Brazil, Colombia, Nicaragua and Bolivia) were significantly lower than high-income countries, again consistent with estimates from efficacy studies [6, 48].

Our systematic review found substantial differences in the quality and design of studies and considerable heterogeneity. However, there was no evidence of publication bias. Heterogeneity was dealt with by stratifying the analysis by World Bank income classification when heterogeneity was low to moderate and by using sensitivity analysis to identify factors which may have caused bias in the overall estimate. We conclude that the best pooled estimates are provided by stratifying by World Bank country classification. Sensitivity analyses for RVGE hospitalisations and or ED attendances did not identify any substantial effects resulting from differences in study quality. Exclusion of studies from countries with only state based or private provision of RV vaccination made little difference to the overall effect estimates.

Sub-analyses by vaccine dose identified that 1 dose of RV vaccine conferred a lower overall VE estimate than full course dose, particularly in middle income countries, indicating that there is a clear benefit for children completing the full schedule. Wider confidence intervals were seen for partial dose estimates due to smaller available sample sizes which are likely a result of the majority of children completing recommended schedules.

It was difficult to assess VE by age as the different studies reported VE for different age groups and therefore finding standard categorisations for age was problematic. Pooled VE estimates in children aged >1 year of age in middle income countries were lower than for that for infants <1 year of age. This could be due to the higher relative disease burden in infants, a consequence of acquiring natural immunity with age, independent of vaccination [49]. Nonetheless, classifications used here showed no significant differences between age groups in both high and middle income countries, suggesting that the RV vaccination is highly effective against RVGE hospitalisation regardless of age. Interestingly, one study from Colombia reported high VE in the <1 year olds (84.4% VE; 95% CI 23-97%) but negative VE in >1 year olds (-79% VE; 95% CI -556 to 51%), the authors indicated that this could be because of a low sample size in the older group and a change in the predominant rotavirus strains circulating in Colombia during the study to heterotypic non-vaccine strains [29].

Additionally, whilst prior to vaccination the predominant strain type in many high income countries was G1P [8, 50] in lower and middle income countries there is often greater strain diversity and concurrent circulation of several strains simultaneously [1, 50]. It is possible that the frequency of more strains that heteroptypic to vaccine types may contribute to the lower effectiveness of the vaccine in middle income countries. However, there are likely to be inherent immununological and epidemiological factors at play [51].

Using meta-analysis to review VE against community consultations for AGE is particularly important in high income countries where the majority of the healthcare burden from rotavirus infection is in community healthcare settings. For instance in the UK rotavirus was deemed to be responsible for approximately 800 000 general practice consultations per year prior to vaccine introduction [4]. Pooled estimates presented here show a considerably lower vaccine effectiveness against AGE community consultations compared with RVGE hospitalisations in high income countries. Since only one study is available for middle income countries, evaluation of the effectiveness of vaccination against AGE community consultations in this setting is difficult, particularly because this study had high risk of bias predominantly due to significant differences in age of the vaccinated and unvaccinated cohorts.

Whereas clinical trials have suggested lower efficacy against milder disease [48, 52], a single study here reported VE against laboratory confirmed RVGE community consultations on par with VE against RVGE hospitalisations [20, 21]. Whilst there was no-indicator of disease severity in the study, the healthcare setting suggests milder disease. Therefore there is a need for more robustly designed studies in middle income settings and high income countries in order to properly assess RV vaccine effectiveness against milder disease resulting in community consultations for RVGE.

Studies reviewed here represent countries with a World Bank country classification of high or middle income, and clearly show lower VE in middle income countries. However, the burden of disease is likely to be greater in middle income countries representing a superior population greater potential benefit of vaccination in these settings. At time of review no studies could be included from low income settings However, findings from a recent study in Malawi suggest a VE of 64% (95% CI 24–83), similar to that reported in middle income countries [53]. Future reviews will be required to capture studies from these settings.

As these studies were conducted in the “real world” with population level vaccine introduction the VE estimates are likely to include both the direct effect and any herd protective effect of population vaccination. This could be particularly significant in cohort studies with high population vaccine uptake. Indeed only one study attempted to separate the herd protection and direct effect from the overall effect of vaccination, estimating a substantial increase in indirect effect of vaccination as cohort vaccine uptake increased over time [30]. More evidence of the indirect effect of vaccination is therefore required through subsequent cohort studies.

We searched three widely used databases—PubMed, Web of Science, and Academic Search Premier —as well as grey literature using a pre-specified, systematic search protocol. We were able to quality assess the studies included using an established critically appraised tool specifically for use with non-randomised studies in meta-analysis, allowing a good understanding of a studies validity importantly with reference to RCT as a gold standard. The majority of studies were assessed as being at low or moderate risk of bias strengthening the meta-analysis for RVGE hospitalisation. However, our assessment of study bias used one specific tool, the Newcastle-Ottawa Scale, with some author defined criteria, therefore it is possible that another bias assessment tool and criteria would identify different risk of bias. Furthermore, variations in study outcome definitions and statistical methods could have introduced error into some of our meta-analyses. For instance, heterogeneity was moderate to high in some of the meta-analyses, particularly those that examined community consultations; this could be related to varying definitions of a community consultation.

## Conclusions

This review and meta-analysis has enabled the systematic production of pooled VE estimates for rotavirus vaccination globally from the literature. We conclude that RV vaccines represent a highly effective preventive measure against severe rotavirus disease, with “real world” vaccine effectiveness estimates as high as efficacy measures from RCTs. There is sufficient evidence to promote the continued roll out of both RV vaccines in both high and middle income settings. The modest benefits from vaccination against community consultations for RVGE represent information which can be used in appropriate cost-effectiveness studies, which may provide better understanding of the value of reducing mild to moderate disease through vaccination.

## Additional file

Additional file 1: Table S1. Study characteristics of seven cohort studies published between January 2006 and April 2014. **Table S2.** Study characteristics of twenty three case-control studies published between January 2006 and April 2014. (DOCX 32 kb)

## Abbreviations

ARI: Acute respiratory infection
CI: Confidence interval
ED: Emergency department
ICD: International Classification of Diseases
NOS: Newcastle-Ottawa Scale
OR: Odds ratio
PRISMA: Preferred Reporting Items for Systematic Reviews and Meta-Analyses
RCT: Randomised controlled trials
RR: Relative risks / risk ratios
RV1: Rotarix® vaccine
RV5: RotaTeq® vaccine
RVGE: Rotavirus gastroenteritis; acute gastroenteritis
VE: Vaccine effectiveness
WHO: World Health Organisation
