# Supplementary material for: Population effectiveness of the pentavalent and monovalent rotavirus vaccines: a systematic review and meta-analysis of observational studies
Source: BMC Infect Dis. 2017 Aug 15;17:569. doi: 10.1186/s12879-017-2613-4 (PMC5556361; doi:10.1186/s12879-017-2613-4)
Supplement: Additional file 1: Table S1. — Study characteristics of seven cohort studies published between January 2006 and April 2014. Table S2. Study characteristics of twenty three case-control studies published between January 2006 and April 2014. (DOCX 32 kb) [file 12879_2017_2613_MOESM1_ESM.docx]

**Table S1 study characteristics of seven cohort studies published between January 2006 and April 2014.**

| **Study** | **Country** | **Vaccine introduction year** | **Vaccine coverage** | **Study period** | **Setting** | **Outcome** | **Cohort definition** | **Vaccine status** | **VE measure** |
| --- | --- | --- | --- | --- | --- | --- | --- | --- | --- |
| Eberly et al. 2011 | USA | 2006 | 54% | July 2003-June 2009 | Military health system database | Hospitalisation for RVGE | Cohort of all US military dependents <5 years enrolled in Department of Defence’s health care program | Outpatient records contained within database | 1-RR x 100 (crude) |
| Fontes-Vieira et al. 2011 | Brazil | 2006 (National immunisation programme) | NR | December 2006-December 2008 | Monitored at home every 2 weeks | All-cause diarrhoea and RV (+) diarrhoea | Cohort of 500 children under 1 year. Reports cumulative incidence of all-cause diarrhoea and number of samples RV positive in vaccinated and unvaccinated groups. | Vaccination card and health centre databases | NR calculated by reviewers. |
| Muhsen et al. 2011 | Israel | 2007 (partial reimbursement offered) | 55% | September 2008-January 2009 | Health maintenance organisation (HMO) database | AGE requiring a physician visit in infants < 1 year (Physician diagnoses coded as AGE according to ICD 9) | Cohort of 34,642 infants analysed. Exposure variable: RV vaccine purchased before September 1^st^ 2008 | Vaccine purchases (HMO database) | 1-RR x 100, stratified by number of doses purchased and socio-economic status |
| Nolan et al. 2012 | USA (Philadelphia) | 2006 | 78.2% (1+) 65% (full) | Feb 2006 – Feb 2008 | Electronic Health Record of a Paediatric Practice Based Research Network | AGE community consultation, AGE after hours telephone calls, or AGE episode (combination of calls and consultations occurring within 10 days of each other to estimate discrete episodes) | A total of 24,679 children eligible for RVV.  Cohort 1 - children eligible in both 2007 & 2008 – 13951 (9351 received vaccine). Further divided in to 1a – 2007 season, and 1b 2008 season (mutually exclusive) Cohort 2 – children only eligible in 2008 – 10728 (9958 received vaccine). | Electronic Health Record | 1-IRR x 100, adjusted for age at start of season, race, practice location, presence of a chronic condition, well child visits up to date, non-RV immunisations up to date, total sick-child visits, time in cohort. |
| Panozzo et al. 2014 | USA | 2006 | 51% in 2007 - 86% I n 2010 | Born May 2000-April 2005 and born May 2006- April 2010 | National Health Insurance Claims Database | ICD9 Codes identifying RVGE & AGE hospitalisations | Cohort of 905,718 children aged 8-20 months who had received at least 1 dose of DTaP. | Coding in National Health Insurance Claims Database | 1-Hx100 Cox regression. Age was the time variable and analyses were stratified by year and adjusted for birth month. |
| Wang et al. 2010 | USA | 2006 | NR | 2007 & 2008 Rotavirus Seasons | National Health Insurance Claims Database | ICD10 Codes identifying RVGE & AGE outpatient consultations, hospitalisations and ED presentations. ED presentations and hospitalisations were combined into one outcome. | A total of 42306 infants who had received at least one dose of RV5 and a concurrent group of 28,417 infants who had not received RV5 but had received a first dose of DTaP. | Vaccination codes or National drug codes o health insurance claims. | VE = 1- rate ratio comparing infants receiving RV5 to DTaP. AGE outcomes were adjusted for gender and calendar year. RVGE outcomes not adjusted due to small numbers |
| Wang et al. 2013 | USA | 2006 | NR | 2007 & 2008 Rotavirus Seasons | National Health Insurance Claims Database | ICD10 Codes identifying RVGE & AGE outpatient consultations, hospitalisations and ED presentations. ED presentations and hospitalisations were combined into one outcome. | A total of 33140 infants who had received a full course of RV5 and a concurrent group of 26167 infants who had not received RV5 but had received a full course of DTaP | Vaccination codes or National drug codes o health insurance claims. | 1- RR x 100 AGE outcomes were adjusted for gender and calendar year. RVGE outcomes not adjusted due to small numbers |

**Table S2 study characteristics of twenty three case-control studies published between January 2006 and April 2014.**

| **Study** | **Country** | **Vaccine introduction year** | **Vaccine coverage** | **Study period** | **Setting** | **Outcome** | **Case definition** | **Control definition** | **Vaccine status** | **VE measure** |
| --- | --- | --- | --- | --- | --- | --- | --- | --- | --- | --- |
| Case-Control studies | | | | | | | | | | |
| Bellido-Blasco et al. 2012 | Spain (Castellon) | 2006 (privately available) | 21.8% (control group) | 2009 | Laboratory surveillance | Laboratory detection | Children 2-35 months of age with Diarrhoea and laboratory (+) RV. Mixed infections excluded | Children 2-35 months of age with AGE with laboratory (-) RV | Immunisation registry | 1-OR*100 adjusted for age, hospitalisation and time of year. Logistic regression. |
| Braeckman et al. 2012 | Belgium | 2006 (National immunisation programme, partially reimbursed) | >90% | February 2008-June 2010 | Random sample of 39 hospitals | Hospitalisation for RVGE | Children with AGE aged 3-59 months with laboratory (+) RV | Non-AGE controls – matched to case’s DOB attending hospital or outpatient clinic. | Vaccination card or medical record | 1-mOR*100 from logistic regression adjusting for sex, medical history, attendance at day care, maternal breast feeding, maternal education, attendance at preschool and household size |
| Carvalho-Costa et al. 2009 | Brazil (Rio de Janeiro) | 2006 (National immunisation programme) | 58% (control group) | February 2005 to December 2007 | A paediatric hospital | Hospitalisation for RVGE | Children<60 months of age with AGE and dehydration requiring IV fluid replacement with laboratory (+) RV | Children<60 months of age with AGE and dehydration requiring IV fluid replacement with laboratory (-) RV | Unknown | 1-OR*100 (crude OR calculated by review team) |
| Castilla et al. 2012 | Spain (Navarre) | 2006 (privately available) | 18% (control group) | January 2008- June 2011 | Health service database | RVGE or AGE health care contact or Hospitalisation | Children with AGE aged 3-59 months with laboratory (+) RV | Children with AGE aged 3-59 months with laboratory (-) RV | Immunisation registry | 1-OR*100, adjusting for age group, sex, birth year, major chronic conditions, health care setting and area |
| Correia et al. 2010 | Brazil | 2006 (National immunisation programme) | >50% | March 2006 - September 2008 | A paediatric hospital | Hospitalisation or ED visit for RVGE | Children under 60 months of age with severe diarrhoea defined as treatment with IV fluid replacement with laboratory (+) RV | Two groups 1) Children under 60 months of age with severe diarrhoea defined as treatment with IV fluid replacement with laboratory (-) RV. 2) Children hospitalised with ARI | Vaccination card | 1-OR *100 unconditional logistic regression adjusting for month and year of birth |
| Cortese et al. 2011 | USA (Minnesota, Georgia and Connecticut) | 2006 | In controls 41-63% fully vaccinated | December 2006 – June 2007, December 2007- June 2008, December 2008 - June 2009 | 5 Hospitals | Hospitalisation or ED visit for RVGE | Children 56days and older with AGE laboratory (+) RV | Two groups:  1) Children with AGE with laboratory (-) RV. 2) Matched controls from Immunisation registry. Matched on Zip code and birth date | Hospital providers or immunisation registry | 1-OR*100 adjusting for site, season and birth quarter. Exact unconditional logistic regression |
| Cortese et al. 2013 | USA (Georgia and Connecticut) | 2006 | In controls 72% fully vaccinated with RV1 | January 2010-June 2010 and January 2011-June 2011 | 5 Hospitals | Hospitalisation or ED visit for RVGE | Children >7 months of age with AGE laboratory (+) RV | Two groups:  1) Children with AGE with laboratory (-) RV. 2) Matched controls from Immunisation registry. Matched on Zip code and birth date | Hospital providers or immunisation registry | 1-OR*100 adjusting for site, season and birth quarter. Exact unconditional logistic regression |
| Cotes-Cantillo et al. 2014 | Colombia | 2009 (Expanded programme of immunisation) | >90% | January 2009 - January 2011 | Health centres with EDs in six cities | Hospitalisation or ED visit for RVGE | Children aged <60 months with diarrhoea and laboratory (+) RV. | Children aged <60 months with diarrhoea and laboratory (-) RV. | Vaccination card | 1-OR*100 adjusting for age and birth quarter, dehydration, and vomit. Unconditional logistic regression |
| de Palma et al. 2010 | El Salvador | 2006 | In controls 85% | Jan 2007 to June 2009 | Seven hospitals based in cities | Hospitalisation for RVGE | Children under 60 months of age with dehydration with laboratory (+) RV | For each case three controls from the community were matched on case date of birth | Vaccination card or vaccination registry | 1-OR *100 conditional logistic regression adjusting for sex, medical history, attendance at day care, maternal breast feeding and SES |
| Desai et al. 2010 | USA (Connecticut) | 2006 | In controls 30% | March 2006 - July 2009 | A paediatric hospital | Hospitalisation for RVGE | Children 8 weeks to 3 years of age with laboratory (+) RV | Two group 2 matched controls per group: 1) Hospitalised children with AGE (-) RV or hospitalised for non-AGE. Matched on date of birth and date of hospitalisation. 2) Non-hospitalised children registered at the same medical centre as case. Also matched for date of birth. | Medical records | 1-mOR*100 from logistic regression adjusting for sex, race, ethnicity, day-care attendance, breast feeding, chronic illness, premature birth, income and tobacco exposure. |
| Donauer et al. 2013 | USA (Rochester, Cincinnati, Nashville) | 2006 | 74% (≥ 1 dose) | December 2006 – June 2007 and December 2007- June 2008 | Prospective active population based surveillance at 3 sites | Hospitalisation or ED visit for RVGE | Lab-confirmed rotavirus in children < 3 years | Three groups:  1) Representative sub-cohort of children registered with primary care practices.  2) Children with AGE negative for RV;  3) Children with acute respiratory infection. (2&3 at same institutions as cases and matched by date of birth) | Immunisation records, immunisation registries and review of medical charts | (1) 1-HR*100 adjusted for DOB, insurance status, breast feeding and days spent at risk; (2&3) 1-OR*100, adjusted for age, breastfeeding, insurance status and site |
| Guh et al. 2011 | USA (Connecticut) | 2006 | In controls 22% at least partially vaccinated | July 2006-December 2008 | 2 Paediatric specialty hospitals | Hospitalisation for RVGE | All infants aged ≥ 2 months but < 3 years with laboratory (+) RV | No hospitalisation for RV in study period. Matched by birth date and residence | Connecticut immunisation registry and tracking system | 1-mOR*100 conditional logistic regression |
| Ichihara et al. 2014 | Brazil | 2006 (National immunisation programme) | In controls 90% at least partially vaccinated | July 2008-August 2011 | National RV Acute Diarrhoea Surveillance System | Hospitalisation for RVGE | Children aged 4-24 months admitted with acute diarrhoea and (+) RV. Hospital stay at least 24 hours and first hospitalisations only. | Hospital controls recruited from same hospital as cases. No previous history RV-A diarrhoea and no vaccine preventable disease. Frequency matched for age and sex. | Vaccination card | 1-OR*100, adjusting for sex and age, year of birth and robust variance estimation of Jackknife, with clusters being hospitals |
| Justino et al. 2011 | Brazil (Belém) | 2006 (National immunisation programme) | 85% partially vaccinated (Community controls) | May 2008-May 2009 | Active surveillance at 4 large paediatric hospitals | Hospitalisation for RVGE | Children at least 12 weeks of age hospitalised with lab-confirmed severe RVGE | Two groups:  1Community and 1 hospital control without gastroenteritis per case. Matched by birth date. | Vaccination card | 1-OR*100,adjusting for potential confounders including recruitment period, underlying medical conditions, diet and breastfeeding) |
| Martinon-Torres et al. 2011 | Spain (not reimbursed) | 2008 | 40% | October 2008-June 2009 | Paediatric research network including primary, ED and hospital settings | Any episode of RVGE and hospitalisation for RVGE | Children under 2 years seeking care due to AGE with laboratory (+) RV | Children with AGE with laboratory (-) RV | Vaccination record | 1-OR*100 (crude) |
| Mast et al. 2011 | Nicaragua | 2006 (National immunisation programme) | 92% partially vaccinated (Community controls) | February 2007-October 2009 | Prospective active RV surveillance programme at 6 hospital sites | Hospitalisation or ED visit for RVGE | Severe (Vesikari score ≥ 11) wild type RVGE in children under 5 years | Two groups  1) Community controls, age and residence matched.  2) Hospital controls, acute non-diarrhoeal infectious disease, age-matched | Child health cards, health centre records if cards not available | 1-OR*100, Final model adjusted for income, potential confounders included in univariate analysis included maternal education, gender, Maternal employment; mothers age, income, breastfeeding birth weight, premature. |
| Muhsen et al. 2011 | Israel | 2007 (partial reimbursement offered) | In controls 36% at least partially vaccinated | November 2007-December 2009 | Active surveillance at 3 hospitals in Northern Israel | Hospitalisation for RVGE | Children born August 2007 or later hospitalised with laboratory (+) RV | Children hospitalised with diarrhoea with laboratory (-) RV | Parents’ report | 1-OR*100, adjusting for season, age, hospital, socio-economic status, birth year and month |
| Patel et al. 2009 | Nicaragua | 2006 | In controls 88% were at least partially vaccinated. | 2007-2008 | 4 hospitals in Nicaragua | RVGE requiring overnight admission (other outcome measures included but not reported here) | Children age-eligible to receive RV5 who were admitted with diarrhoea and laboratory (+) RV | Two groups:  1) Community - homes to left and right of case visited until 3 age matched controls identified 2) Hospital - children seeking care at ED or outpatient clinic, unrelated to diarrhoea or vaccine preventable illness, and matched to DOB within 30 days. | Obtained from parent and considered confirmed if vaccination card or clinic records completed. | VE = 1 –mOR x 100 Unadjusted findings presented, as adjusting for potential confounders did not change results Confounders tested included gender, underlying chronic illness, breastfeeding, day-care attendance, maternal education, no. of children, household size and socioeconomic status required to change estimate by more than 10%. |
| Patel et al. 2012 | Nicaragua | 2006 | <1 Year 79% average had 1 dose over period of study.  35% average had 1 dose over period of study | 2007-2010 | 4 community hospitals in Nicaragua | Hospitalisation with diarrhoea, laboratory (+) RV (other outcomes e.g. IV hydration – not reported here) | Children age-eligible to receive RV5 vaccine presenting with acute diarrhoea laboratory (+) RV. | Three groups: 1) Non-diarrhoea controls – matched to case’s DOB from 2 sources – hospital and community. Hospital -were seeking care at ED or clinic or admitted to same hospital as the case, with an illness unrelated to diarrhoea or a vaccine preventable condition.  2) Community controls were found by visiting homes to left and right of case home until 3 controls identified. 3) Children hospitalised with diarrhoea laboratory (-) RV | Obtained from parent and considered confirmed if vaccination card or clinic records completed. | VE = 1-OR x 100 VE calculations adjusted for month of birth, age at hospitalisation, and hospital. Confounders tested included gender, underlying chronic illness, breastfeeding, day-care attendance, maternal education, no. of children, household size and socioeconomic status required to change estimate by more than 10%. |
| Patel et al., 2013 | Bolivia | 2008 | National coverage 76% 2010, 80% 2011 | March 2010 – June 2011 | Six hospitals in Bolivia | Hospitalisation with diarrhoea, laboratory (+) RV (other outcomes considered but not presented here). | Children admitted overnight with acute diarrhoea testing positive for RV, eligible to receive at least one dose of RV1, | Two groups: 1) Hospital Controls – children admitted to same hospital for acute illness unrelated to diarrhoea or a vaccine preventable condition, eligible to receive at least 1 dose of RV1, with a DOB within 30 days of case. 2) Children hospitalised with diarrhoea lab-negative for RV | Obtained from parent and considered confirmed if vaccination card or clinic records completed. | 1-adjusted OR x100 non-diarrhoea controls matched on age and hospital, and adjusted for gender, number of children and rooms at home, a computer at home. Test negative controls adjusts for age in months, month/year of birth, gender, hospital, number of children and rooms in home, computer at home. |
| Payne et al. 2013 | USA | 2006 | In controls fully vaccinated with:  RV1 in 2010 46%; 47% in 2011: RV5 53% in 2010 and 63% in 2011 | November 2009 – June 2011 | Range of surveillance hospital sites in USA. | Rotavirus disease presenting to ED or requiring hospitalisation, age-eligible for vaccination. | Children <5 years of age visiting ED or hospitalised with AGE laboratory (+) RV | Those children enrolled in the study who were found to be laboratory (-) RV | Contact with subject’s primary care provider and regional immunization systems. | VE = (1-OR) x 100  Presented stratified analysis across a range of factors. Adjusted for insurance status and clinical setting but results not presented. |
| Snelling et al. 2011 | Australia | 2006 | In controls 72% fully vaccinated | March – July 2011 | Medical record review of all children admitted to Alice Springs hospital during an outbreak | ICD10 codes for infectious gastroenteritis in medical records. Subgroup of those RVGE positive. | All children aged <5 admitted to Alice Springs Hospital with gastroenteritis during an outbreak, with ICD10 codes for infectious gastroenteritis | Retrospectively conducted matched controls from a record of central Australian births registered on hospital information database. | Vaccination determined from central immunisation database. | VE = 1-OR x 100 (Cases and controls matched for indigenous status and date of birth (within 7 days). Adjusted for remote residence. |
| Staat et al. 2011 | USA | 2006 | In controls 54% any dose | 2007-2009 rotavirus seasons | Prospective surveillance conducted in 3 US counties as part of New Vaccine Surveillance Network | Hospitalisations and ED visits for RVGE in children attending the surveillance hospitals during the rotavirus seasons. | All children attending the ED or hospitalised with AGE with laboratory (+) RV | Two groups:  1) Children with AGE with laboratory (-) RV. 2) ARI controls: children with ARI symptoms who were residents of same study county. | Parents documentation of vaccination. If not available, obtained from state registries. | VE = (1-OR) x 100 Cases were matched to controls according to DOB and symptom onset date. Adjusted for insurance status and clinical setting. |

VE= vaccine effectiveness; RVGE=rotavirus gastroenteritis; AGE=acute gastroenteritis; RR= relative risks / risk ratios; IRR=incidence rate ratio; ARI= acute respiratory infection; ED= emergency department; (+) RV = laboratory confirmed positive rotavirus; (-) RV = laboratory confirmed negative rotavirus; mOR = matched Odds Ratio; DTaP = diphtheria, tetanus, acellular polio; RV1= Rotarix vaccine; RV5= RotaTeq vaccine; HR= Hazard rate ratio.
